# One Step Purification—Vaccine Delivery System

**DOI:** 10.3390/pharmaceutics15051390

**Published:** 2023-05-01

**Authors:** Ernesto R. Soto, Charles A. Specht, Chrono K. Lee, Stuart M. Levitz, Gary R. Ostroff

**Affiliations:** 1 Program in Molecular Medicine, UMass Chan Medical School, Worcester, MA 01605, USA; 2 Department of Medicine, UMass Chan Medical School, Worcester, MA 01605, USA

**Keywords:** glucan particles, nickel nanoparticles, histidine-tagged proteins, vaccine

## Abstract

Glucan particles (GPs) are hollow, porous 3–5 µm microspheres derived from the cell walls of Baker’s yeast (*Saccharomyces cerevisiae*). Their 1,3-β-glucan outer shell allows for receptor-mediated uptake by macrophages and other phagocytic innate immune cells expressing β-glucan receptors. GPs have been used for the targeted delivery of a wide range of payloads, including vaccines and nanoparticles, encapsulated inside the hollow cavity of GPs. In this paper, we describe the methods to prepare GP-encapsulated nickel nanoparticles (GP-Ni) for the binding of histidine (His)-tagged proteins. His-tagged Cda2 cryptococcal antigens were used as payloads to demonstrate the efficacy of this new GP vaccine encapsulation approach. The GP-Ni-Cda2 vaccine was shown to be comparable to our previous approach utilizing mouse serum albumin (MSA) and yeast RNA trapping of Cda2 in GPs in a mouse infection model. This novel GP-Ni approach allows for the one-step binding of His-tagged vaccine antigens and encapsulation in an effective delivery vehicle to target vaccines to antigen-presenting cells (APCs), antigen discovery, and vaccine development.

## 1. Introduction

The development of safe and effective vaccines is needed for a broad range of diseases, including malaria, HIV, cryptococcosis, and emerging highly pathogenic arboviruses (e.g., chikungunya and Zika viruses). There are three main approaches to vaccine development, which are as follows: (1) the traditional approach that employs immunization with inactivated or live-attenuated infective agents; (2) immunization with subunits, such as recombinant proteins and carbohydrate antigens; and (3) the recent development of COVID-19 vaccines using mRNA encapsulated in PEGylated liposomes. The mRNA and subunit vaccine approaches offer advantages over inactivated or live-attenuated vaccines that do not contain the pathogen or its genetic material. However, these approaches are generally less efficacious and require the use of adjuvants, the production and purification of the antigens, and effective vaccine delivery vehicles [1].

The production of recombinant protein antigens in *Escherichia coli* with a six-histidine (6-His) tag at the amino or carboxyl terminus of the protein is commonly used to enable purification by affinity chromatography. Poly-histidine has a strong affinity for divalent metals (Zn^2+^, Co^2+^, Ni^2+^, and Cu^2+^), and affinity resins are commercially available for the purification of His-tagged proteins. These resins generally consist of agarose functionalized with metal chelators, such as nitrilotriacetic acid (NTA), that exhibit high binding constants for metal ions, such as nickel (II) and cobalt (II). The quadridentate NTA ligand forms a highly stable complex with the metal while leaving two binding sites available for tight but reversible His-tagged protein binding [2]. Proteins lacking the His tag can be easily removed by washing the column with solutions containing high salt concentrations, non-ionic detergents, or reducing agents. The His-tagged protein is then eluted with imidazole (up to 500 mM at pH 8.0), EDTA (up to 100 mM) [2,3], or by elution with a low pH gradient (6.0 to 4.0) in the presence of denaturing agents (e.g., urea or guanidine HCl) [4]. The purified recombinant proteins are typically dialyzed free of low-molecular-weight materials and can then be formulated as a subunit vaccine. Other novel approaches have been reported for screening and purification of His-tagged proteins employing matrices, such as nickel-decorated graphene nanoparticles [5], nickel (0)/nickel (II) hydroxide nanoparticles [6], and nickel (0)/nickel (II) oxide nanoparticles [7]. However, these approaches are generally limited to improving antigen screening or purification tools. A one-step His-tagged antigen purification/screening/delivery vehicle could represent a paradigm shift in vaccine development by significantly reducing the time and cost for antigen identification and the preparation of subunit vaccines.

Glucan particles (GPs) derived from Baker’s yeast have been used for the efficient encapsulation of a wide range of molecules and targeted payload delivery to macrophages and dendritic cells [8,9,10,11,12,13,14,15,16,17,18,19,20,21,22,23,24]. The hollow and porous nature of GPs (3–5 µm in diameter) allows for the absorption and retention of the payload molecules. Their β-1,3-D glucan surface composition also provides for receptor-targeted delivery via recognition by cell surface receptors in macrophages and other phagocytic innate immune cells via dectin-1 (D1) and complement receptor 3 (CR3) [20,25]. We have reported several methods for the efficient encapsulation of a variety of payloads, including water-soluble macromolecules (e.g., DNA, siRNA, and proteins) encapsulated inside GPs by both polyplex and layer-by-layer (LbL) synthetic approaches [8,9,23], neutral, small drug molecules encapsulated by embedding the payload in polymer hydrogels [18], and encapsulation within small nanoparticles prepared in situ inside GPs or loaded onto the surface of GPs in a piggyback approach [21,22].

GPs have also been optimized as a vaccine delivery platform through the encapsulation of antigens and adjuvants using polyplex and LbL synthetic approaches [10,11,12,13,15,23,24] and, recently, through developing an approach to encapsulate the proteins in a thermostable silica cage formed in situ within the cavity of GPs [26]. We previously provided proof of principle for a vaccination approach that packages fungal antigens against cryptococcosis in GPs [15,24]. Developing a vaccine against cryptococcosis is needed given the high prevalence of this disease in patients with immunosuppressed systems and its associated morbidity and mortality (~150,000 cases annually in HIV-infected people with a ~74% mortality rate and a 1–5% lifetime risk of infection in solid organ transplant recipients [27]). Soluble antigens from alkaline extracts of mutant cryptococcal strains that have reduced levels of capsule and chitosan or individual recombinant protein antigens were loaded onto GPs and complexed with mouse serum albumin (MSA) and yeast RNA (yRNA). Our previous studies showed that mice treated with antigens encapsulated in GPs mounted antigen-specific CD4+ Th1 and Th17 cell responses and were protected from subsequent lethal challenges with *C. neoformans* [24].

Here, we describe a new application of GPs as a combined protein purification and vaccine delivery platform using GP-encapsulated nickel nanoparticles (GP-Ni) for the binding of His-tagged labeled proteins. Nickel nanoparticles were synthesized in situ within the hollow GP cavity, and a GP-Ni formulation was directly used for the binding of a His-tagged Cda2 cryptococcal antigen previously shown to be protective against cryptococcosis [24]. The GP-Ni-Cda2 vaccine was shown to be comparable to our previous approach utilizing MSA-yRNA trapping of Cda2 in GPs in a mouse infection model. This novel approach allows for the one-step purification of His-tagged vaccine antigens from *E. coli* lysates or in vitro transcription/translational products and encapsulation in an effective delivery vehicle to target vaccines to antigen-presenting cells (APCs), antigen discovery, and vaccine development.

## 2. Materials and Methods

### 2.1. Materials

The glucan particles were prepared from Fleischmann’s Baker’s yeast (AB Mauri Food Inc., Chesterfield, MO, USA), as previously described [8]. The Cda2 gene from *C. neoformans* was synthesized and cloned into pET19b (GenScript Biotech, Piscataway, NJ, USA) so that the vector-encoded His tag was integrated with the N terminus of the cDNA. The recombinant protein was made in the *E. coli* strain BL21(DE3) (New England Biolabs, Ipswich, MA, USA) and purified on His·Bind resin (EMD Millipore, Burlington, MA, USA), as previously described [24]. All of the reagents, solvents, and buffer solutions were purchased from Sigma-Aldrich (Burlington, MA, USA), unless specified otherwise, and used without further purification. The tissue culture materials were purchased from Gibco (Waltham, MA, USA).

### 2.2. Synthesis and Characterization of Glucan Particle–Nickel Nanoparticles (GP-Ni)

Synthesis of GP-Ni: Nickel sulfate was absorbed into GPs by swelling a dry GP pellet in a sub-hydrodynamic volume (5 µL/mg GP) of an aqueous nickel sulfate hexahydrate solution at room temperature. The loaded GPs were then lyophilized, and the loading and lyophilization steps were repeated until the target concentrations of nickel sulfate within the GP samples were achieved (the target concentrations varied from 0 to 1116 µg Ni/mg GP). The dry GP-NiSO_4_ pellets were then treated by wetting the pellets in a sub-hydrodynamic volume (2 µL/mg GP) of sodium borohydride (600 mg/mL) solution in water (Caution: sodium borohydride reacts exothermically with water, generating hydrogen gas. The sodium borohydride solution must be made immediately before use, and the preparation of this solution and the addition to GP NiSO_4_ should be performed in a fume hood). The sodium borohydride solution (2 µL/mg GP) was added two more times with 15–20 min of incubation between each addition. The samples were incubated at room temperature for one hour after the final aliquot was added to the sample to allow for the complete consumption of the sodium borohydride. The samples were washed three times with water to remove small nickel nanoparticles (<30 nm) not trapped within the GPs. The GP-Ni concentration was then adjusted to 2.5 × 10^9^ GPs/mL in water, and the samples were stored at −20 °C.

Spectrophotometric quantification of nickel: The GP-Ni samples (0.25 mg GP) were incubated in 5 M sulfuric acid (100:1 H_2_SO_4_:Ni molar ratio) for 24 h at room temperature. The samples were centrifuged, and the supernatants containing soluble NiSO_4_ were collected. The pH was adjusted to pH 6 with 10 M sodium hydroxide. The samples (180 µL) were mixed with 20 µL of 100 mM 2-mercaptoethanol in 100 mM borate buffer (pH 9) in a 96-well plate. The absorbance of the nickel–2-mercaptoethanol complex was measured at 405 nm to quantify the amount of nickel trapped in the GPs using a calibration curve prepared with nickel sulfate hexahydrate reacted with 2-mercaptoethanol.

Cytotoxicity assay: To determine the effect of nickel on cell growth and viability, the GP-Ni samples and empty GP controls were evaluated with J774 immortalized murine macrophage cells (J774A.1; ATCC Catalog TIB-67). The particles were evaluated for particle cell uptake at a 10:1 GP:cell ratio to maximize the phagocytic cell uptake. The samples were suspended in complete DMEM (10% heat-inactivated FBS, 5% L-glutamine, and 0.1% ciprofloxacin) and added to 96-well plates containing 1 × 10^4^ cells/well. The plates were incubated at 37 °C under 5% CO_2_ for 24 h. Alamar blue (10 µL; 0.22 mg/mL) was added and incubated at 37 °C for 30 min, and the Alamar blue fluorescence was measured using a Tecan Safire 2 (Tecan, Männedorf, Switzerland) plate reader (excitation wavelength = 530 nm; emission wavelength = 590 nm). The fluorescence response is dependent on the reduction of the Alamar blue indicator by metabolically active cells and is a measure of cell number and viability. The growth arrest was calculated from the fluorescence response of the sample relative to the normalized response of the positive control cells (100% viability) containing DMEM and buffer (PBS) and the negative control (0% viability) cells lysed with 0.1% Triton X-100.

Inding of Cda2 to GP-Ni: The GP-Ni samples were washed with protein binding buffer (0.5 M NaCl, 5 mM imidazole, and 20 mM Tris buffer; pH 7.9) and then incubated in a solution of Cda2 protein (50 µg Cda2/mg GP) in binding buffer containing 4 M urea. The samples were incubated at room temperature for 6 h. The pellets were centrifuged to collect the unbound protein (supernatant fraction), and the pellet was washed once with PBS. Cda2 was extracted following incubation of GP-Ni-Cda2 in eluting buffer (binding buffer containing 4 M imidazole, 6 M urea, and 25 mM EDTA) for 4 h at room temperature. The Cda2 protein in the unbound (supernatant), wash, and bound (pellet) fractions was monitored by SDS-PAGE with Coomassie blue staining. This binding experiment allowed for the evaluation of GP-Ni samples with varying ratios of µg Ni per mg GP to select the GP-Ni sample with the highest binding capacity for Cda2. The selected GP-Ni sample was scaled up for the preparation of a GP-Ni-Cda2 vaccine.

### 2.3. Vaccination and Challenge Studies

The GP-Ni-Cda2 vaccine was prepared at a target protein concentration of 10 µg Cda2/200 µg GP, and the sample was diluted to a GP concentration of 5 mg GP/mL (2.5 × 10^9^ GP/mL; 1 mg GP ~ 5 × 10^8^ GPs). A control GP-Cda2-yRNA vaccine was prepared at the same protein-to-GP ratio following the methods previously described for GP vaccines encapsulating protein/yRNA polyplexes [23]. The GP-encapsulated Cda2 protein was extracted from the GP-Ni-Cda2 samples by incubation in eluting buffer (4 M imidazole, 6 M urea, 25 mM EDTA, and 20 mM Tris buffer; pH 7.9) and from the GP-Cda2-yRNA samples by incubation in SDS-PAGE loading dye containing 6 M urea, as previously described [23]. The amount of Cda2 bound to the GP-Ni and control vaccines was quantified by SDS-PAGE with Coomassie blue staining.

The GP-based vaccines (0.1 mL dose; 5 mice per group) were administered to C57BL/6 mice (The Jackson Laboratory, Bar Harbor, ME, USA) three times at two-week intervals as a subcutaneous injection at the midline of the abdomen. Two weeks following the third vaccination, the mice were challenged with the *C. neoformans* strain KN99. The mice were anesthetized with 2% isoflurane (Piramal Healthcare, Mumbai, India) in a laboratory animal anesthesia system (VetEquip, Livermore, CA, USA) and inoculated oratracheally with 50 µL of fungal cell suspension in PBS to deliver 1 × 10^4^ colony-forming units (CFUs) of Cryptococcus cells. For the survival studies, the mice were monitored twice daily for morbidity. At the termination of the study, the surviving mice were euthanized, and their lungs were removed and homogenized in 4 mL of PBS containing penicillin and streptomycin. The undiluted and diluted homogenates were plated on Sabouraud dextrose agar and incubated at 30 °C for 2–3 days, at which time the CFUs of Cryptococcus were enumerated. The animal care and procedures were in accordance with protocols approved by the UMass Chan Medical School Institutional Animal Care and Use Committees.

All graphs and statistical analysis were generated using GraphPad Prism, version 9.3.1. The Kaplan–Meier survival plots were evaluated using the Mantel–Cox test for multiple pairwise comparisons.

## 3. Results and Discussion

Glucan particles have been utilized for phagocyte receptor-mediated targeted delivery of a wide range of payloads, including DNA, RNA, protein, and small drug molecules [8,9,10,11,12,13,14,15,17,18,19,20,21,22,23,24]. In previous work applying GP technologies to vaccine delivery, we have developed methods to encapsulate antigen proteins by non-covalent trapping of proteins within a mouse serum albumin (MSA)–yeast RNA (yRNA) complex [23] or by encapsulation in a thermostable silica cage formed in situ within the cavity of GPs [26]. Additionally, we are currently evaluating the application of these GP encapsulation methods for the preparation of mRNA vaccines.

In this paper, we show that GPs can be used to prepare in situ encapsulated nickel nanoparticles, allowing for the binding of His-tagged proteins and vaccine production. Most commercially available nickel-based purification columns contain crosslinked agarose with nitrilotriacetic acid (NTA) ligands for the binding of nickel ions onto the resin. We first evaluated the synthetic approaches for the preparation of GPs encapsulating a polymer derivatized with NTA. As an example, alginate (Alg) can be chemically modified to link NTA ligands to a fraction of the carboxylic acid groups. The derivatized Alg-NTA can then be loaded onto GPs, and the remaining free carboxylic acid groups can be used for crosslinking with calcium chloride to form an encapsulated Ca-Alg-NTA gel. The caveats with this approach are the need for multiple synthetic steps and low His-tagged protein binding capacity. The samples of GP-(Ca-Alg-NTA) were prepared with a maximum binding capacity of 28 µg Ni/mg GP, which was insufficient to bind 50 µg Cda2 protein/mg GPs.

**Figure 1:**
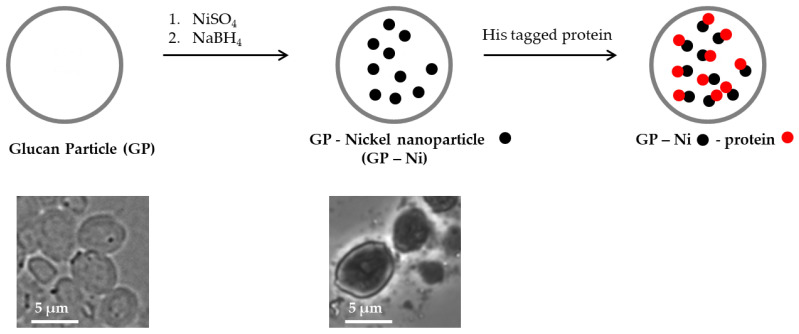
Schematic representation of the synthesis of glucan particle-encapsulated nickel nanoparticles (GP-Ni) for His-tagged protein binding, and microscopy pictures showing empty GPs and GP-Ni samples.

We developed a more synthetically feasible approach in which nickel was encapsulated in glucan particles by the in situ synthesis of nickel nanoparticles through the reduction of nickel sulfate inside the GPs with sodium borohydride, as depicted in Figure 1.

The selected method to synthesize GP-encapsulated nickel nanoparticles in situ is a rapid one-step reaction that is compatible with the need to employ aqueous-based solutions for efficient absorption into GPs. It is known that nickel salts treated under mild conditions with sodium borohydride as a reducing agent yield mixtures of nickel metal and nickel boride nanoparticles, boron byproducts B_2_O_3_, and borate (B_2_H_6_) [28]. The presence of Ni (II) species in the nanoparticles in the form of nickel boride is required as the primary binding site for histidine. A detailed analysis of the composition of the nickel species prepared in situ in the GPs is beyond the scope of this current work. The GP-Ni samples were prepared with the objective of demonstrating the feasibility of binding His-tagged protein antigens inside GP-Ni and antigen delivery to APCs to generate an effective immune response.

**Figure 2:**
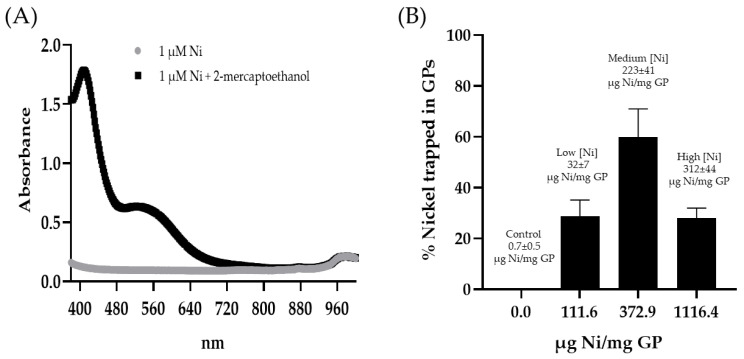
(**A**) Absorbance spectrum of nickel sulfate (1 µM Ni) with and without addition of 100 mM 2-mercaptoethanol in borate buffer. The formation of a nickel complex with 2-mercaptoethanol increases the absorbance response; the linear range for the detection of nickel by the measurement of absorbance at 405 nm is from 20 to 100 µM without the addition of 2-mercaptoethanol and from 0.2 to 1 µM with 2-mercaptoethanol. (**B**) Nickel encapsulation efficiency in GPs at three target µg Ni/mg GP ratios quantified by the measurement of absorbance at 405 nm of a nickel/2-mercaptoethanol complex after the extraction of nickel from the GPs. The calculated µg Ni/mg GP for each sample is shown above each bar in the graph (the results show the average of three GP Ni samples).

GP-Ni samples at three Ni/GP weight ratios were synthesized with the goal of identifying reaction conditions to prepare GP-Ni samples that provide the following: (1) maximum encapsulation efficiency of Ni; (2) maximum binding of His-tagged Cda2 proteins; and (3) minimal cytotoxicity. The GP-Ni samples were synthesized and then digested in sulfuric acid to extract the nickel for spectrophotometric quantification of the nickel complex with 2-mercaptoethanol [29]. The optimized GP in situ reduction of nickel sulfate with sodium borohydride yielded a maximum encapsulation efficiency of 60% at a concentration of 372.9 µg Ni/mg GP (Figure 2). The reduction of nickel sulfate with sodium borohydride generates nanoparticles with a heterogeneous size distribution. At a lower nickel concentration (111.6 µg Ni/mg GP), it is likely that a high proportion of small (<30 nm) Ni NPs were generated and lost by diffusion out of the GPs during washing, resulting in low yields. Aggregation of the nanoparticles at the optimal Ni concentration likely reduced this loss. At higher nickel concentrations (1116 µg Ni/mg GP), the GP cavity is likely saturated with nickel sulfate and sodium borohydride, preventing efficient reactions inside the particles and subsequent nickel sulfate reduction outside the GPs and loss during the post-synthesis washing steps.

**Figure 3:**
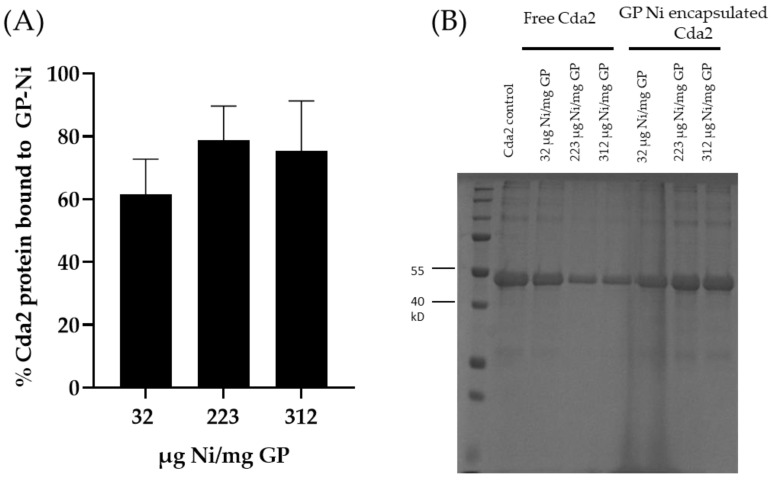
(**A**) Encapsulation efficiency of Cda2 protein (50 µg Cda2/mg GP) in GP-Ni samples (average of three measurements) and (**B**) SDS-PAGE showing free and encapsulated Cda2 fractions from three GP-Ni samples.

The GP-Ni samples were evaluated for His-tagged protein binding by incubation with His-tagged Cda2 protein at a ratio of 50 µg Cda2/mg GP-Ni. This concentration was selected based on our previous work, in which encapsulating 50 µg of protein in 1 mg of GPs by non-covalent trapping with yRNA yielded highly effective vaccines. The unbound protein fraction was separated from GP-Ni by centrifugation, and the GP-Ni pellets were washed to remove any His-tagged Cda2 proteins not bound to the GP-encapsulated nickel nanoparticles. The pellets were then incubated in an elution buffer containing imidazole and EDTA to reverse the His-tagged binding to the nickel nanoparticles. SDS-PAGE analysis of the different fractions demonstrates binding of the His-tagged Cda2 to GP-Ni (Figure 3). The results of the GP-Ni samples prepared at medium (223 µg Ni/mg GP) and high (317 µg Ni/mg GP) ratios show that ~80% of the Cda2 protein was efficiently trapped in GP-Ni.

**Figure 4:**
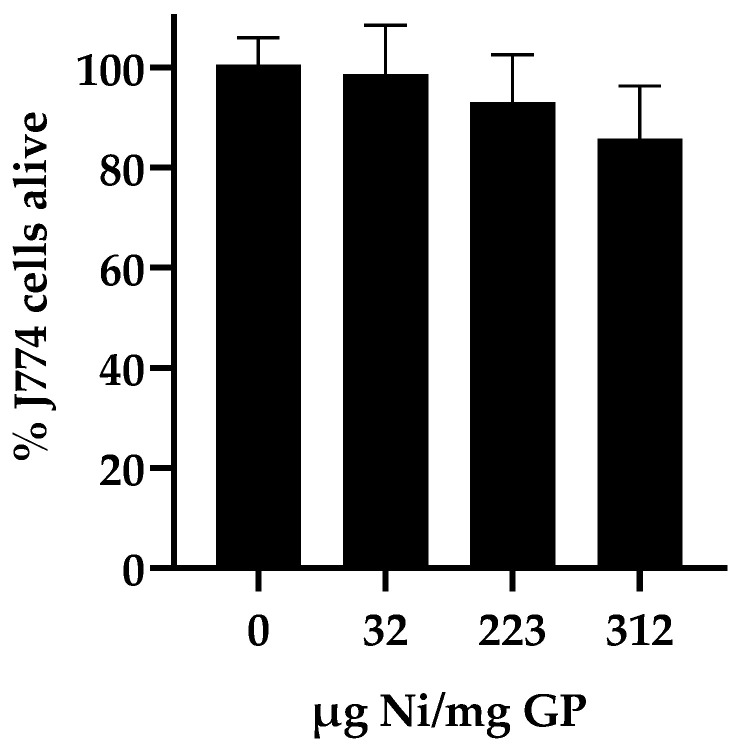
Macrophage (J774 cells) viability after 24 h of incubation with empty GPs and GP-Ni samples. The particle samples were added to the cells at a GP:cell ratio of 33:1; the cell viability was quantified by measuring the Alamar blue fluorescence (the experimental results are shown as the average and standard deviation of three experiments). The control samples of nickel sulfate solution delivering the same concentration of nickel as the GP-Ni samples showed lower cell viability (50–65%).

In a final characterization experiment to select the best GP-Ni sample for the synthesis of a Cda2 vaccine, the GP-Ni samples were evaluated for cytotoxicity in macrophage cells. We have previously shown that empty GPs and GPs encapsulating nanoparticles are nontoxic to cells at particle:cell ratios of up to 33:1 GP:cell [21,22]. We examined the cytotoxicity of the GP-Ni formulations by adding GP-Ni to macrophage cells at a ratio of 33:1 GP-Ni: cell. The samples were incubated for 24 h, and the cell viability was assessed using the Alamar blue viability assay. The results (Figure 4) showed cell viability greater than 80% for all GP-Ni formulations.

**Figure 5:**
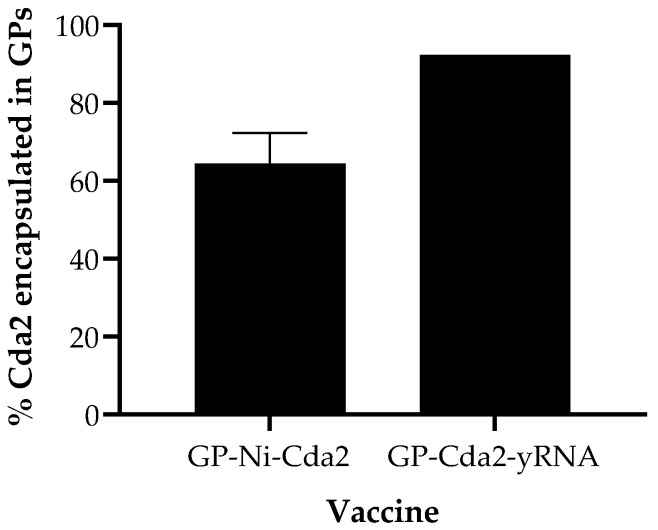
Encapsulation efficiency of Cda2 in the GP-Ni-Cda2 and GP-Cda2-yRNA vaccines used in the in vivo study.

The results of the GP-Ni formulation characterization demonstrated that GP-Ni with a final nickel concentration of 223 µg Ni/mg GP is optimal for the scale-up of in situ synthesis of GP-Ni. The selected GP-Ni formulation showed the following: (i) high nickel-trapping efficiency; (ii) efficient His-tagged protein binding; and (iii) lack of toxicity to macrophage cells. In addition to the GP-Ni-Cda2, a positive control Cda2 vaccine was prepared by encapsulating the protein in GPs through trapping in an MSA-yRNA matrix. This encapsulation approach for the control vaccine has been reported previously to generate vaccines effective at protecting mice against cryptococcal infections [24]. Both the GP-Ni-Cda2 and GP-Cda2-yRNA vaccines were prepared at a target Cda2 concentration of 50 µg Cda2/mg GP (1 mg GP~5 × 10^8^ GPs). The amount of trapped protein was quantified following elution of Cda2 from GP-Ni or extraction from GP-Cda2-yRNA following an extraction method previously reported for GP-protein-yRNA samples [23]. The GP-Ni-Cda2 samples were first incubated in an eluting buffer containing imidazole and urea, and then the samples were extracted in SDS-PAGE binding buffer. The eluting buffer for GP-Ni-Cda2 also contained 10 mM EDTA to maximize the encapsulated protein release from the Ni nanoparticles. Figure 5 shows the Cda2 encapsulation efficiency for the GP samples used in the in vivo vaccine study.

A proof-of-concept vaccine study was conducted to demonstrate the bioactivity of the GP-Ni-Cda2 vaccine. The binding of histidine to nickel is reversible by reaction with excess imidazole or by lowering the pH from the optimal pH of 7–8 for His binding to nickel to an acidic pH value. The release of Cda2 from GP-Ni-Cda2 in vivo is likely to occur by breaking of the Ni-His chelating bond in the acidic environment of lysozymes, as described for His-tag protein delivery vehicles using NTA-Ni-lipid nanoparticles [30], or through proteolysis of the antigen in antigen-presenting cell lysosomes, as previously demonstrated for the release of antigens from GP-antigen-yRNA vaccines [12].

**Figure 6:**
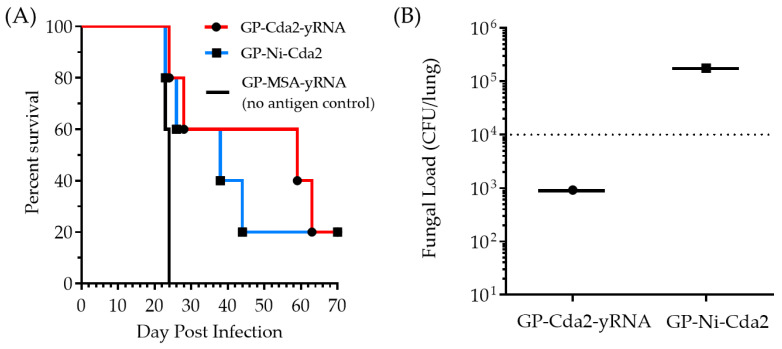
(**A**) Kaplan–Meyer survival curves of mice treated with GP-Cda2 -yRNA, GP-Ni-Cda2, or GP-MSA-yRNA (no antigen control) formulations. The mice vaccinated with GP-MSA-yRNA (no antigen control) were compared to those vaccinated with either GP-Cda2-yRNA or GP-Ni-Cda2 (n = 5 mice per group). Statistical analysis by Mantel–Cox tests showed that survival was significantly different between the groups vaccinated with a Cda2 formulation and the group vaccinated with the no antigen control (*p* < 0.02); the differences between the vaccinated groups were not significant (ns; *p* = 0.64). (**B**) Fungal load from the lung homogenates obtained from the surviving mice euthanized 70 days post-infection (the dotted line represents the inoculum).

The mice were subcutaneously administered with the GP-based vaccines (GP-Ni-Cda2 and GP-Cda2-yRNA) or a GP-MSA-yRNA control and challenged with 10^4^ CFU of the *C. neoformans* strain Kn99. This strain of *C. neoformans* causes a progressive, lethal pulmonary infection in unvaccinated mice [11,15,16,31]. The mice were monitored following the vaccination without any unusual inflammation occurring at the injection sites. The overall behavior of the mice was unchanged following the vaccination, and there were no discernable differences between the mice receiving the GP-MSA-yRNA (no antigen control) or the GP Cda2 vaccines. The mice treated with GP-Ni-Cda2 or GP-Cda2-yRNA showed a similar percent survival (60%) up to 26 days after infection. The GP-Cda2-yRNA vaccine showed a slightly greater effect on protecting the mice than GP-Cda2-Ni after day 26 (Figure 6A). The GP-MSA control-treated animals exhibited 100% mortality 26 days after infection, demonstrating that protection was not due to the stimulatory effects that β-glucans have on innate and trained immunity [32,33]. The fungal load measured from the lung homogenates of the euthanized mice showed a two-log higher CFU in the samples from the animals treated with GP-Ni-Cda2 than the animals treated with the control GP-Cda2-yRNA (Figure 6B). The slightly lower performance of GP-Ni-Cda2 compared to the control vaccine GP-Cda2-yRNA may be due to lower Cda2 binding (Figure 5) and/or a slower rate of Cda2 in vivo release. Nevertheless, this animal study demonstrates the potential of using GP-Ni-Cda2 as a new tool for protein binding and delivery of the GP vaccine technology. Future work will focus on the characterization of nickel species and the optimization of the reaction conditions for more efficient protein binding, toxicological studies of optimized GP Ni compositions (e.g., oxygen stress, cytokine release, and DNA strand break assays), and evaluation of GP-Ni protein purification of His-tag proteins directly from cell lysates for use as vaccines.

## 4. Conclusions

GP-encapsulated nickel nanoparticles were prepared in situ by the reaction of nickel sulfate loaded onto GPs with sodium borohydride. GP-Ni was evaluated for His-tagged Cda2 binding, and a GP-Ni-Cda2 vaccine was used in a murine model of cryptococcal infection. The GP-Ni-Cda2 was effective at protecting the mice, albeit somewhat less efficiently than the control GP-Cda2-yRNA vaccine. The development of this GP-Ni system offers the potential to expand the applicability of GPs for both affinity purification of proteins and APC-targeted protein delivery for vaccine applications.

## Data Availability

The data presented in this study are available upon reasonable request from the corresponding author.
